# A Fluorinated Polyimide Based Nano Silver Paste with High Thermal Resistance and Outstanding Thixotropic Performance

**DOI:** 10.3390/polym15051150

**Published:** 2023-02-24

**Authors:** Zhenhe Wang, Dong Wang, Chunbo Zhang, Wei Chen, Qingjie Meng, Hang Yuan, Shiyong Yang

**Affiliations:** 1 Aerospace Institute of Advanced Materials & Processing Technology, Beijing 100074, China; 2 The Second Military Representative Office of the Air Force Equipment Department in Beijing, Beijing 100081, China; 3 Key Laboratory of Science and Technology on High-Tech Polymer Materials, Institute of Chemistry, Chinese Academy of Sciences, Beijing 100190, China

**Keywords:** fluorinated polyimide, nano silver paste, thermal resistance, thixotropic properties

## Abstract

Because of high conductivity, acceptable cost and good screen-printing process performance, silver pastes have been extensively used for making flexible electronics. However, there are few reported articles focusing on high heat resistance solidified silver pastes and their rheological properties. In this paper, a fluorinated polyamic acids (FPAA) is synthesized by polymerization of the 4,4′-(hexafluoroisopropylidene) diphthalic anhydride and 3,4′-diaminodiphenylether as monomers in the diethylene glycol monobutyl. The nano silver pastes are prepared by mixing the obtained FPAA resin with nano silver powder. The agglomerated particles caused by nano silver powder are divided and the dispersion of nano silver pastes are improved by three-roll grinding process with low roll gaps. The obtained nano silver pastes possess excellent thermal resistance with 5% weight loss temperature higher than 500 °C. The volume resistivity of cured nano silver paste achieves 4.52 × 10^−7^ Ω·m, when the silver content is 83% and the curing temperature is 300 °C. Additionally, the nano silver pastes have high thixotropic performance, which contributes to fabricate the fine pattern with high resolution. Finally, the conductive pattern with high resolution is prepared by printing silver nano pastes onto PI (Kapton-H) film. The excellent comprehensive properties, including good electrical conductivity, outstanding heat resistance and high thixotropy, make it a potential application in flexible electronics manufacturing, especially in high-temperature fields.

## 1. Introduction

Owning to good adaptability to additive manufacturing process, flexibility, low environmental impact and low cost, printed electronics technologies have been used to fabricate flexible electronic devices such as actuators, sensors, and solar cell and displays during the past few decades [1,2,3,4]. As an indispensable component, conductive pastes play a key role in determining the functionality and reliability of electronic devices. At present, a variety of conductive pastes based on different conductive fillers have been developed, including conductive polymer pastes, copper pastes, silver pastes, gold pastes, carbon nanotubes pastes and graphene pastes [5,6,7,8,9,10]. Compared with other conductive pastes, silver pastes with excellent conductivity and remarkable antioxidant properties balance the contradiction between high performance and low cost, and attract extensive attention in academic and applied fields [11,12,13].

Silver pastes are commonly composed of silver powder, adhesive and diluent, supplemented by a small amount of the third component acted as dispersant, leveling agent, defoamer, moisturizer, thixotropic agent, etc. [14,15,16]. Depending on different types of adhesives, silver pastes can be divided into high temperature sintered silver pastes and low temperature solidified silver pastes. Despite showing exceptional heat resistance and electrical conductivity, sintered silver pastes, usually taking glass powder as the adhesive, possess a high forming temperature (more than 500 °C) [17,18,19], which exceeds the decomposition temperature of the polymer substrate and limits its application in flexible electronics. In contract, solidified silver pastes show a relatively low and wide forming temperature ranging from room temperature to 400 °C according to the different adhesives. The low forming temperature, adjustable electrical conductivity and good flexibility make solidified silver pastes an ideal choice for fabricating flexible electronic devices by multiple printing technologies. Liang developed water-based AgNW inks and fabricated the thin-film transistors (TFT) by fully screen printing. The printed TFT had a yield of 91.7% and an average mobility of 33.8 ± 3.7 cm^2^V^−1^s^−1^ [20]. Zhu developed a maskless and templateless fabricating approach for high-performance flexible transparent electrodes with nano silver pastes by combining electric-field-driven microscale 3D printing and hybrid hot-embossing [21]. The fabricated flexible, transparent electrode exhibits excellent photoelectric properties, remarkable mechanical stability, and environmental adaptability.

At present, polymers used as adhesive for preparing silver pastes mainly include epoxy resin, polyacrylic resin, polyurethane, polymethacrylate, etc. [22,23,24,25]. Due to the low heat endurance of those polymers, the operating temperature of silver pastes derived from those polymer adhesives is usually lower than 200 °C, which cannot meet the demand of some flexible electronics for high temperature manufacturing process and high work temperature. Therefore, it is very urgent to develop polymer-based silver pastes with good heat resistance and suitable forming temperature. As a polymer with the best comprehensive performance, polyimide is widely used in the field of electronic packaging. It is considered as an ideal alternative material for preparing high-temperature silver pastes. Nguyen prepared an Ag nanowire–polyimide composite by the solution blending method and expounded the charge transport mechanism in thermoplastic thermostable materials [26]. Li prepared an Ag nanowires/PI composite and used it to fabricate the highly sensitive flexible pressure sensor [27]. Tung-Lin prepared the conductive silver/photosensitive polyimide (PSPI) nanocomposites by homogeneously mixing the PSPI precursor with silver nanoflakes [28]. The resultant nanocomposites possess excellent heat resistance and high conductivity.

As mentioned above, polyimide/silver pastes possess superior over-all performance, especially high thermal resistance. However, the reported polyimides used for preparing silver pastes usually have poor solubility in organic solvent; even the precursor polyamide acid (PAA) can only be dissolved in finite strongly polar aprotic solvents including N,N-dimethylacetamide, N,N-dimethylformamide, 1-methyl-2-pyrrolidinone, m-cresol. Those solvents are not suitable for the preparation of silver pastes because of the following disadvantages: hazard to human body, corrosivity to common polymer substrates and mask, lack of volatility. Moreover, the electrical conductivity of the reported polyimide/silver pastes is relatively low. In addition, there are almost no reports about the rheological properties of polyimide/silver pastes, which have a key effect on the printing accuracy of silver pastes.

In this article, a fluorinated PAAs is synthesized using 4,4′-(hexafluoroisopropylidene) diphthalic anhydride (6FDA) and 3,4′-diaminodiphenylether (3,4′-ODA) as monomer in the diethylene glycol monobutyl ether (DB) solvent. Furthermore, the prepared PAA resin is mixed with silver powder and ground to obtain silver pastes. The influences of the composition of silver pastes on its rheological and conductive properties are studied in detail. Consequently, the obtained silver pastes show an excellent heat resistance, electrical conductivity and outstanding thixotropy, which is very useful for fabricating the fine circuit and possess a great potential in high temperature resistant flexible electronic field.

## 2. Materials and Methods

### 2.1. Materials

**Figure 1:**
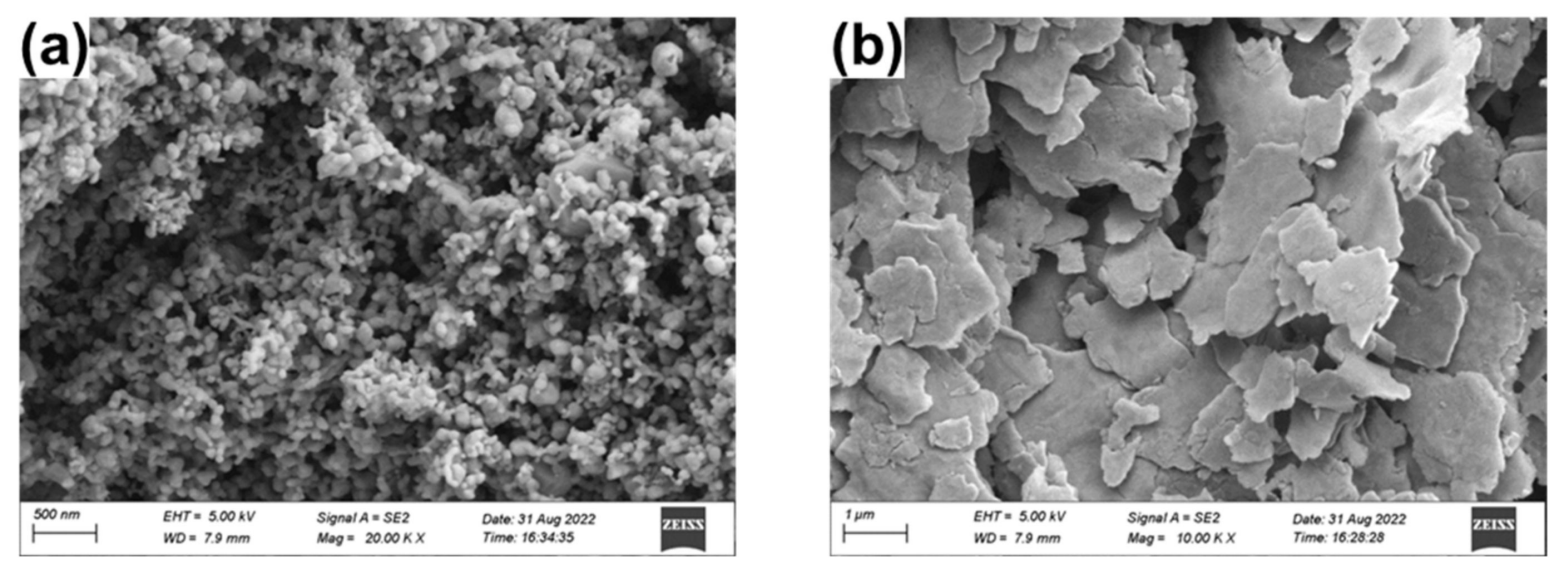
SEM photographs of nano silver powder (**a**) and flake silver powder (**b**).

Nano silver powder (average length of 100 nm) and flake silver powder (average length of 5 μm) were purchased from SINO-PLATINUM METALS CO., LTD and used without pre-treatment. The microscopic morphology of the two silver powders mentioned above is shown in Figure 1. 6FDA, 3,4′-ODA and 4-phenylethynyl phthalic anhydride (PEPA) were supplied by Changzhou Sunlight Pharmaceutical Co., Ltd. 3,4′-ODA was used directly. 6FDA and PEPA should be dried at 160 °C for 12 h prior to use. The defoaming agent (BYK-066N) and flatting agent (BYK-333) were obtained from BYK (China). Diethylene glycol monobutyl ether (DB) was purchased from Aldrich and used without further purification.

### 2.2. Synthesis of Fluorinated Polyamide Acid (FPAA) Resin

**Scheme 1:**
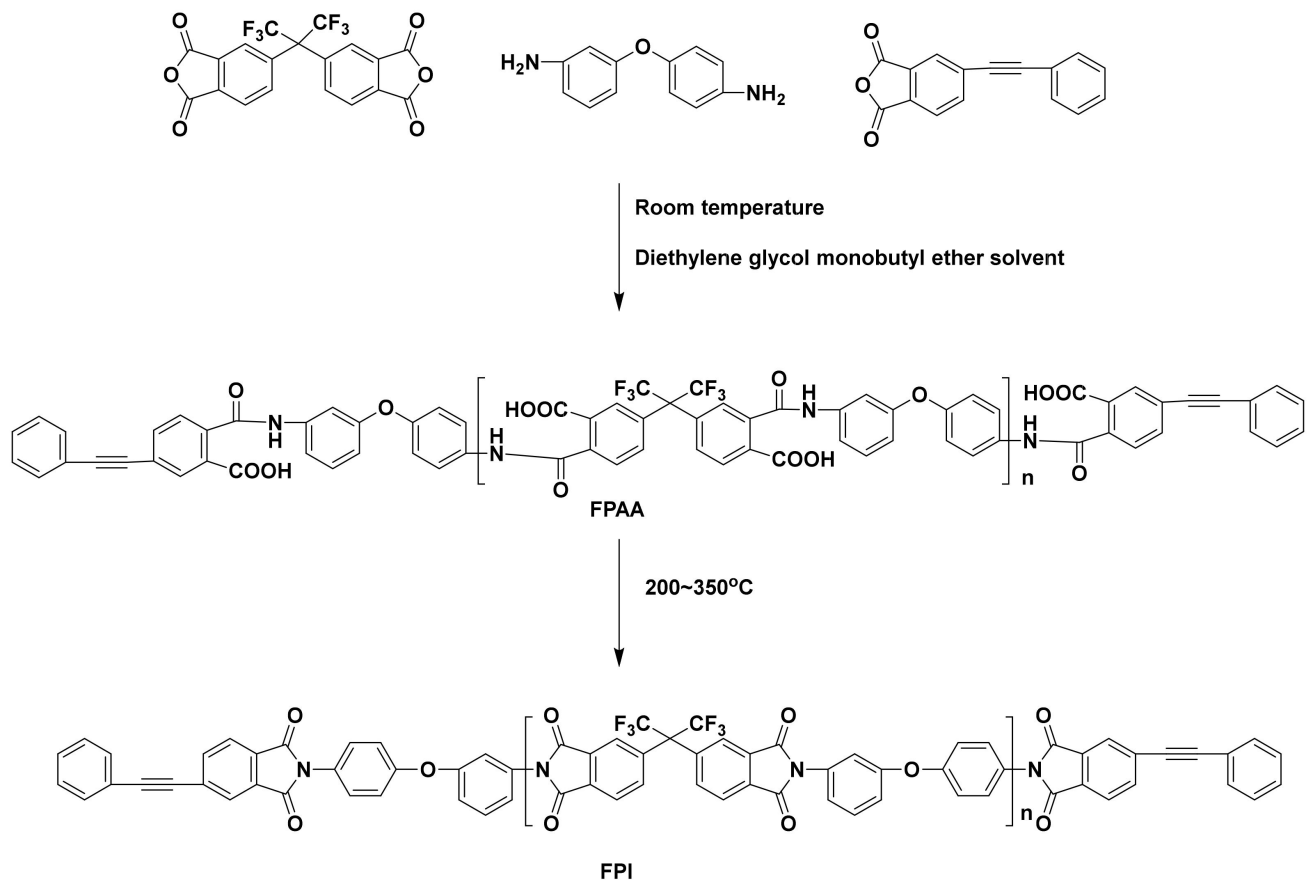
The synthetic route to FPAA resins and FPI.

FPAA resin was prepared from 6FDA, 3,4′-ODA and PEPA in DB solvent by polycondensation reaction as shown in Scheme 1, in which the PEPA was used as a reactive capping agent. Specifically, the synthesis of FPAA resin with solid content of 40 wt% was described. In a 250 mL of three-necked flask equipped with a mechanical stirrer, 3,4′-ODA (5.2279 g, 12.7351 mmol) was dissolved in DB (46.19 mL), 6FDA (2.5000 g, 11.4616 mmol) was then added with mechanical stirring to the solution in one portion. The polycondensation was proceeded at room temperature for 3 h to produce a uniform viscosity resin, in which a stoichiometric amount of PEPA (3.0556 g, 14.0086 mmol) acting as reactive capping agent was added gradually. The mixture was stirred mechanically for 8 h at room temperature to yield a viscous FPAA resin solution, which was sealed and stored at 5 °C.

### 2.3. Preparation of Nano Silver Pastes

Firstly, the prepared FPAA resin and nano-silver powder were mixed by intensively mechanical stirring in the beaker. Secondly, the BYK-333 and BYK-066N were added in obtained slurry acting as flatting agent and defoaming agent respectively. Thirdly, the mixture was finely ground using a three-roller grinder to pulverize the agglomerated particles formed by the nanosilver powder and further facilitate the uniform mixing of each component, to fully obtain highly dispersed nano silver pastes noted as FPI-NSAg-X, where X is 70%, 75%, 80%, 83%, 85% representing the solid percentage content of silver powder in the cured paste. As control group, the silver paste based on flake silver powder as conductive filler was prepared in the above manner and not as FPI-FAg-83%. The detailed compositions of different silver pastes are listed in Table 1.

### 2.4. Measurements

Thermogravimetric analysis (TGA) was performed on TA Q50 thermal analysis system from 40 °C to 760 °C at a heating rate of 20 °C/min under nitrogen atmosphere.

The electrical conductivity of cured silver paste was measured by four-point probe tester (ST2258C, Suzhou Jingge Electronic Co., Ltd., Suzhou, China). The preparation method of the sample to be tested was as follows: the conductive patters were fabricated on the polyimide films (Kapton-H, Wilmington, DE, USA) through screen printing technology and were further treated at different high temperature ranging from 200 °C to 350 °C. The dimensions of the conductive patters were 2 cm × 2 cm.

Rheological behaviors of the formulated silver pastes were carried out on a MCR 92 rheometer (Anton Parr, Graz, Austria) equipped with stainless steel parallel plates with a diameter of 50 mm and gap of 1.0 mm. All rheological properties tests were performed at 20 °C and silver paste need to stand still for 5 min before each test. The shear viscosity tests were conducted with shear rates of 0.01–1000 s^−1^ at a frequency of 1 Hz. The three interval thixotropic test (3ITT) was performed to simulate the screen-printing process with specific test parameters of 0.1 s^−1^ shear rate for 200 s, 100 s^−1^ for 80 s, and 0.1 s^−1^ for 300 s.

Microstructure and the morphology of cured silver paste were examined using Sigma 300 field-emission scanning electron microscope (Zeiss, Oberkochen, Germany).

Resolution of printed conductive pattern and the dispersion of silver pastes were characterized by measuring dimension and morphology of pattern using a DM4 B light microscope (Leica, Wetzlar, Germany).

Adhesion of silver pastes to PI film (Kapton-H) was evaluated by measuring the change of the electrical conductivity of the cured pattern before and after the pulling off experiment.

## 3. Results and Discussion

### 3.1. Dispersion Homogeneity

**Figure 2:**
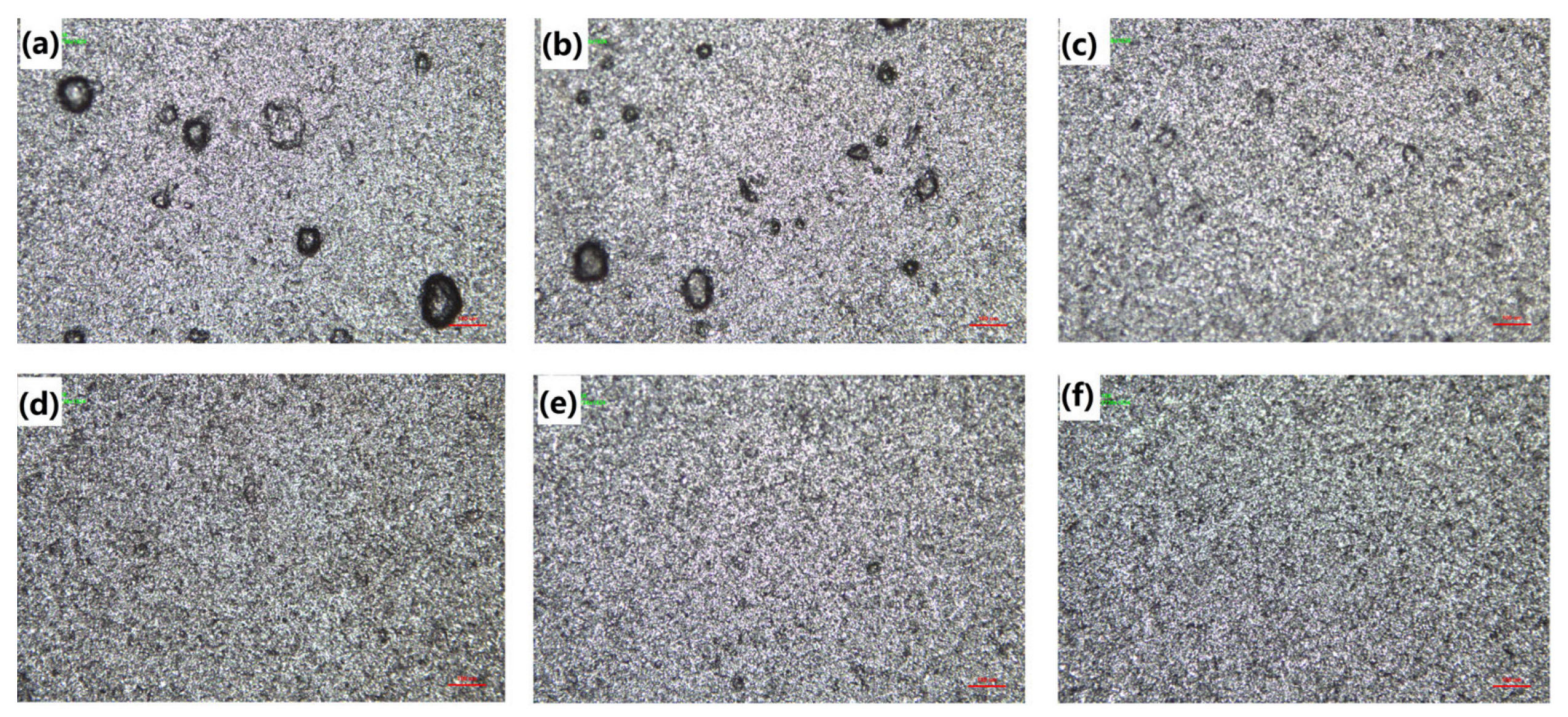
The optical micrograph of nano silver pastes grinded with different roll gaps. (**a**) unground, (**b**) ground with 80 μm, (**c**) ground with 40 μm, (**d**) ground with20 μm, (**e**) ground with 10 μm, (**f**) ground with 5 μm.

**Figure 3:**
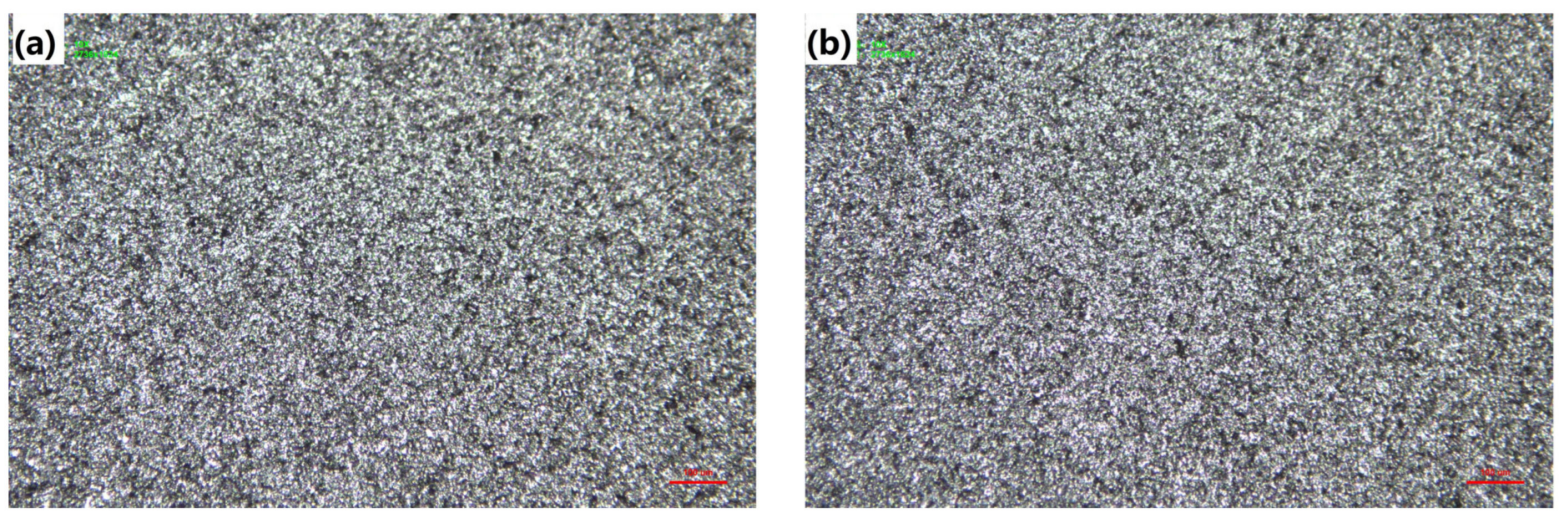
The optical micrograph of nano silver pastes grinded with a roll gap of 5 μm before (**a**) and after (**b**) static storage 3 months.

As multiphase composite system, the dispersion of nano silver powder in polymer binder has a crucial effect on the conductivity, rheology and uniformity of silver pastes. Especially for nano silver pastes, the agglomeration of silver nanoparticles is a very common phenomenon, which is very detrimental to the dispersion of silver powder. In order to break the agglomerated silver nanoparticles and improve the dispersion homogeneity, the mixed nano silver paste was grinded with different gaps by a three-roller grinder. The dispersion of nano silver paste was characterized by high resolution light microscope. Figure 2 show the optical micrograph of nano silver paste (FPI-NSAg-83%) treated by three-roll grinder with different roll gaps. It can be found that the grind method can effectively break the agglomeration of nanoparticles and improve the dispersion uniformity. The number and size of nano agglomerations decrease as roll gaps reducing. When the roll gap is reduced to 5 μm, there is no obvious agglomeration in the field of view. Furthermore, the dispersion uniformity of nano silver paste remains stable within 3 months after static storage. There are no agglomerated particles were found, as shown in Figure 3. It can be explained that the surface of the nano silver powder is coated with polyamic acid resin, which reduces the surface energy of the nano silver powder, thus hindering the occurrence of re-agglomeration.

### 3.2. Electrical Conductivity

To evaluate the electrical conductivity of obtained nano silver paste, the conductive patters with dimension of 2 cm × 2 cm were fabricated on the polyimide films (Kapton-H) through screen printing technology and were further treated at different high temperature ranging from 200 °C to 350 °C. Then, square resistance (*R*_s_) and thickness of conductive patters were measured using four probe tester and steps instrument respectively. The volume resistivity of silver paste was calculated according to Equation (1), where *ρ*_v_ is volume resistivity, W is the thickness of the corresponding conductive patterns.

(1) $$\rho_{v}=R_{s}\times W$$

**Figure 4:**
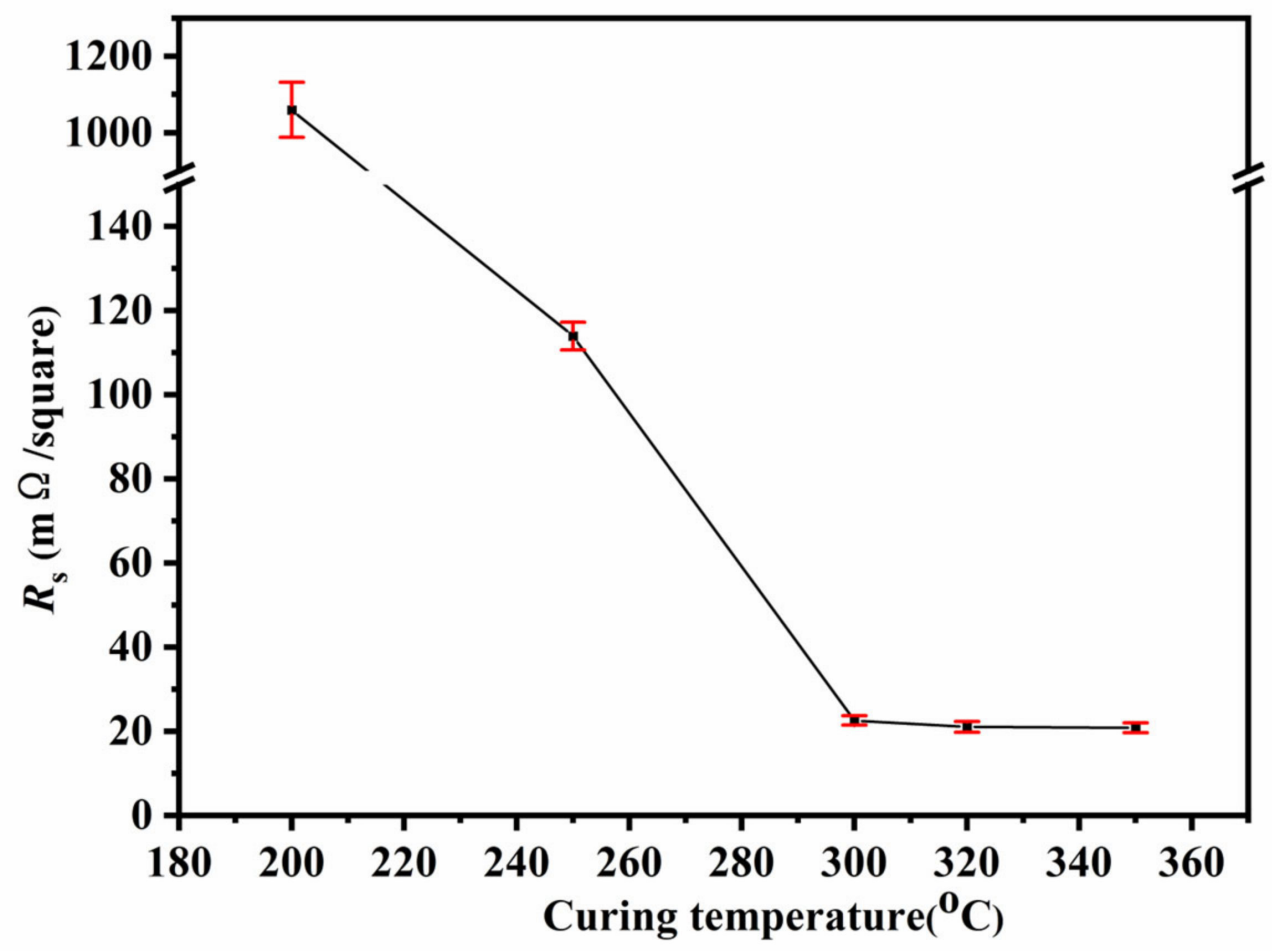
The *R*_s_ of cured silver pastes with different curing temperature.

**Figure 5:**
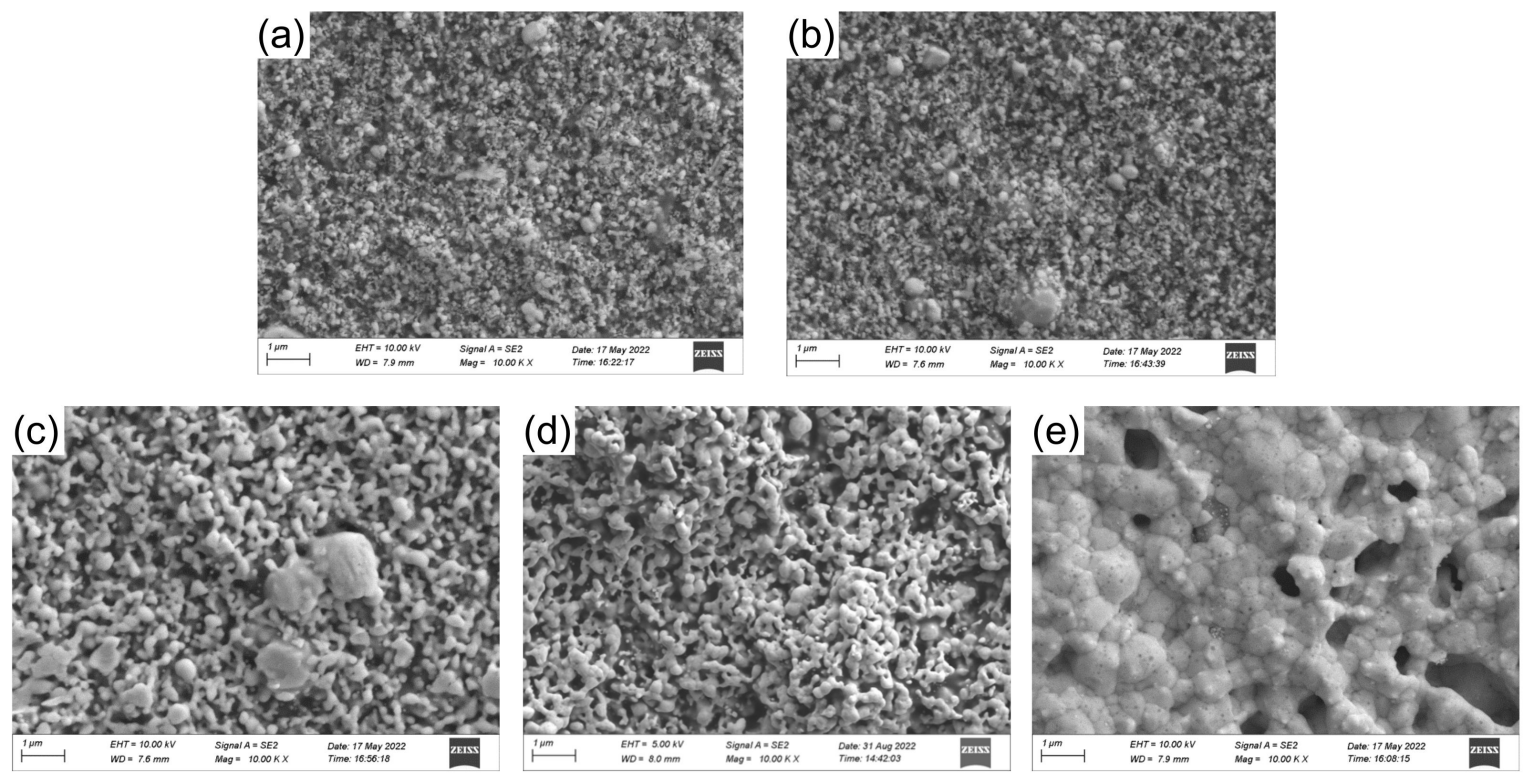
The SEM photographs of conductive pattern at different curing temperatures. (**a**) 200 °C, (**b**) 250 °C, (**c**) 300 °C, (**d**) 320 °C, (**e**) 350 °C.

Figure 4 depicts the *R*_s_ of cured patterns with dimension of 2 cm × 2 cm, which prepared from FPI-NSAg-83% silver paste with different curing temperature. Because the imidization temperature of PAA resin usually exceeds 200 °C, the curing temperature of obtained silver paste is higher than the conventional conductive pastes such as epoxy conductive pastes, acrylate conductive pastes, polyurethane conductive pastes, etc. As shown in Figure 4, with the increase of curing temperature, the *R*_s_ of the conductive patterns decreases significantly, especially when the curing temperature increases to 300 °C, the *R*_s_ decreases to 22.6 mΩ/square. The thickness of pattern is 20 μm and the calculated volume resistivity reaches 4.52 × 10^−7^ Ω·m. When the curing temperature is further increased, the *R*_s_ decreases slowly. As we all known, the diluent and the water produced from imidization reaction will volatilize from pastes as the curing temperature increase, so the mass percentage of silver powder in silver paste increase. Additionally, high curing temperature will promote the in-plane orientation and densification of polyimide molecular chains [29,30,31]. The two reasons mentioned above will reduce the distance between the silver powder and make the silver powder closely connected, which contribute to reducing the resistivity value of the conductive pattern. When the curing temperature reached 300 °C, the imidization reaction and volatilization of diluent in silver paste were completed, the mass percentage of silver powder increased to the highest. Further increasing the curing temperature can also promote the densification of conductive pattern. However, the contribution of this process to elevating the conductivity is negligible comparing the imidization reaction and volatilization of diluent before 300 °C. This speculation can be further confirmed by the microstructure of conductive pattern at different curing temperatures as showed in Figure 5. With the increase of curing temperature, the distance between silver powder particles decreases and the stacking density of silver powder increases. Additionally, when the curing temperature reaches above 300 °C, the nano silver powder partially melts. All of those will contribute to improve the electrical conductivity of the printed pattern at curing temperature of 300 °C.

**Figure 6:**
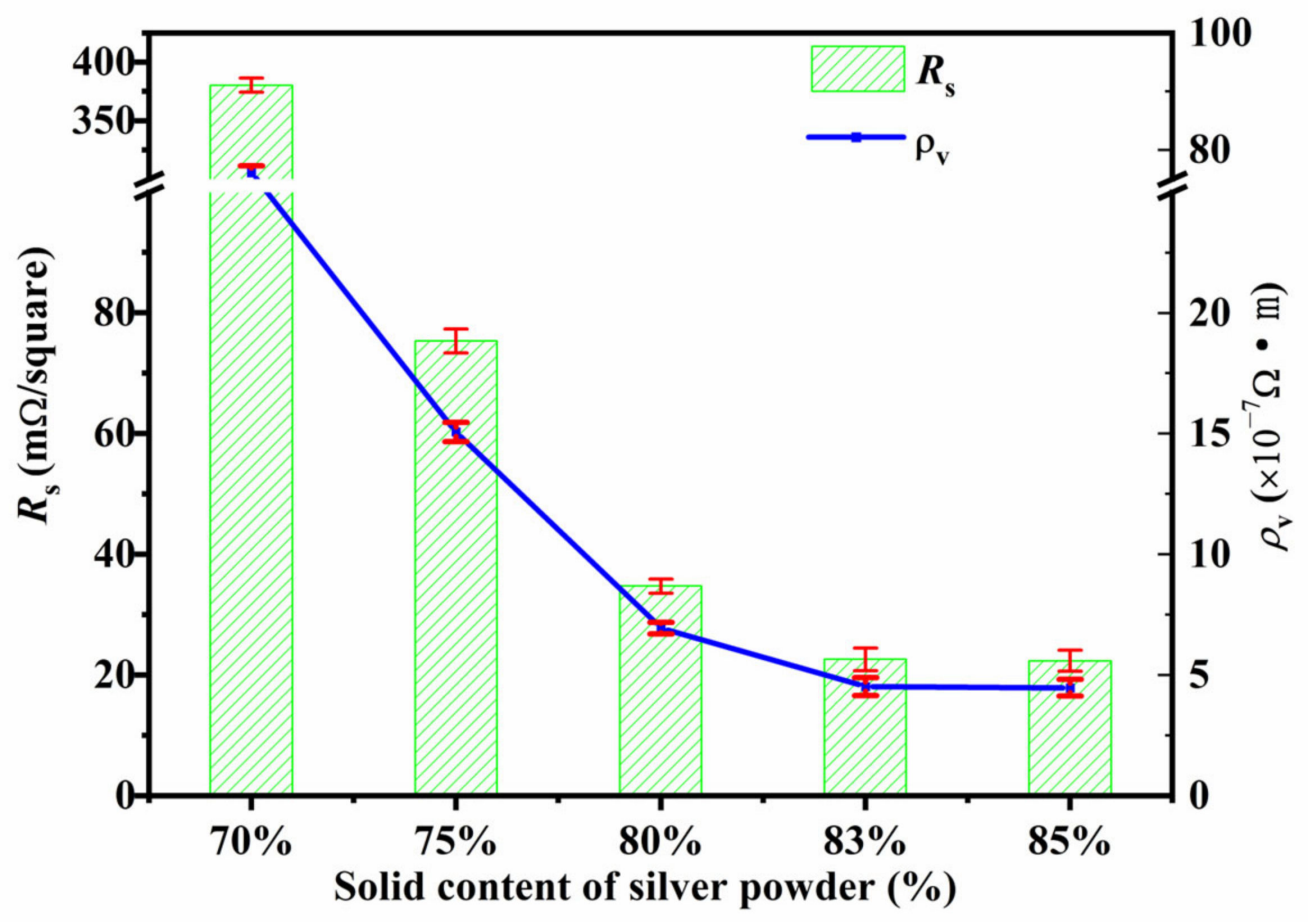
The *R*_s_ and *ρ*_v_ of cured silver pastes with different silver content.

**Figure 7:**
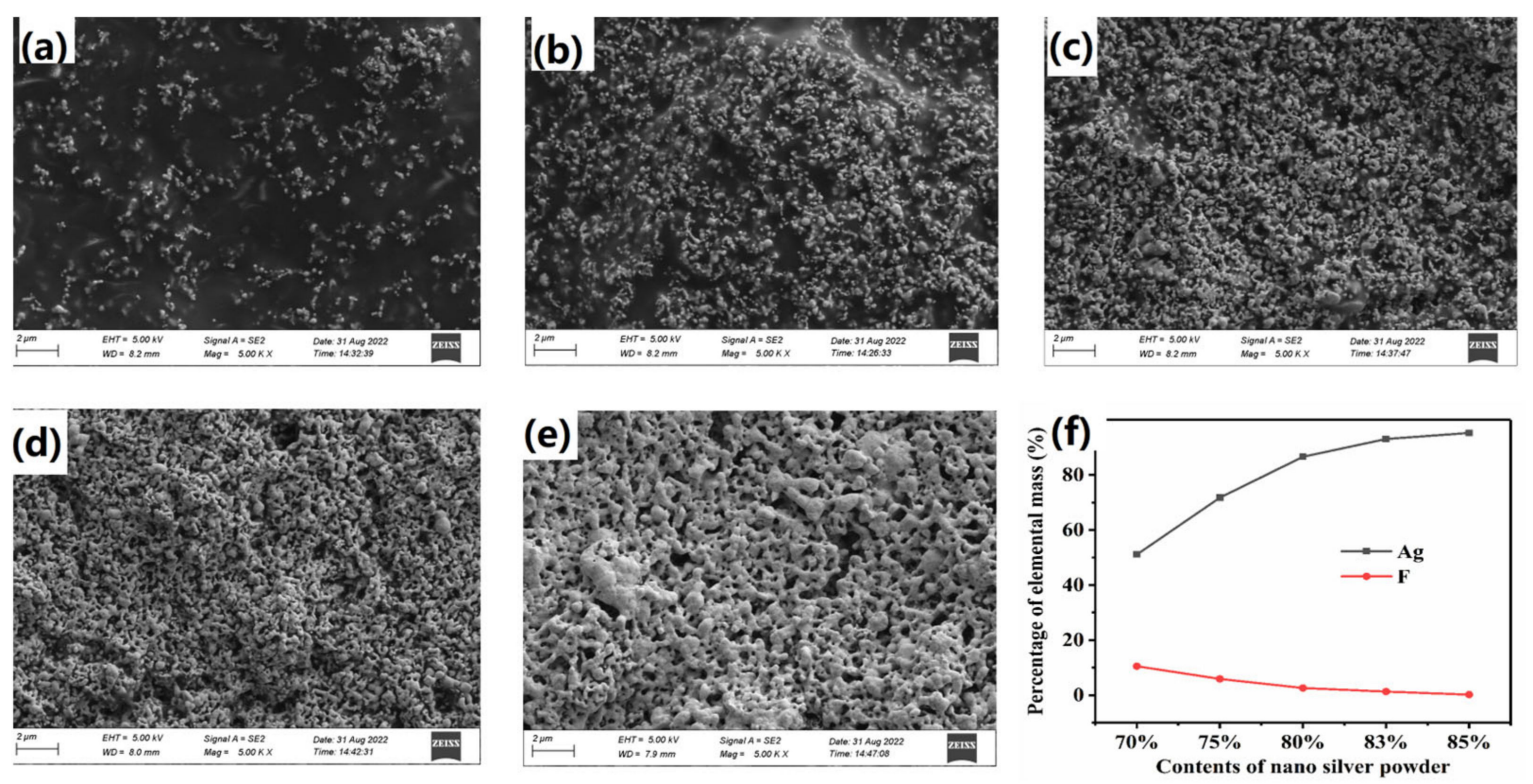
The SEM photography of cured silver pastes with different silver content. (**a**) 70%, (**b**) 75%, (**c**) 80%, (**d**) 83%, (**e**) 85%, (**f**) The content of silver element and fluorine element in different cured silver paste by EDS analyses.

**Figure 8:**
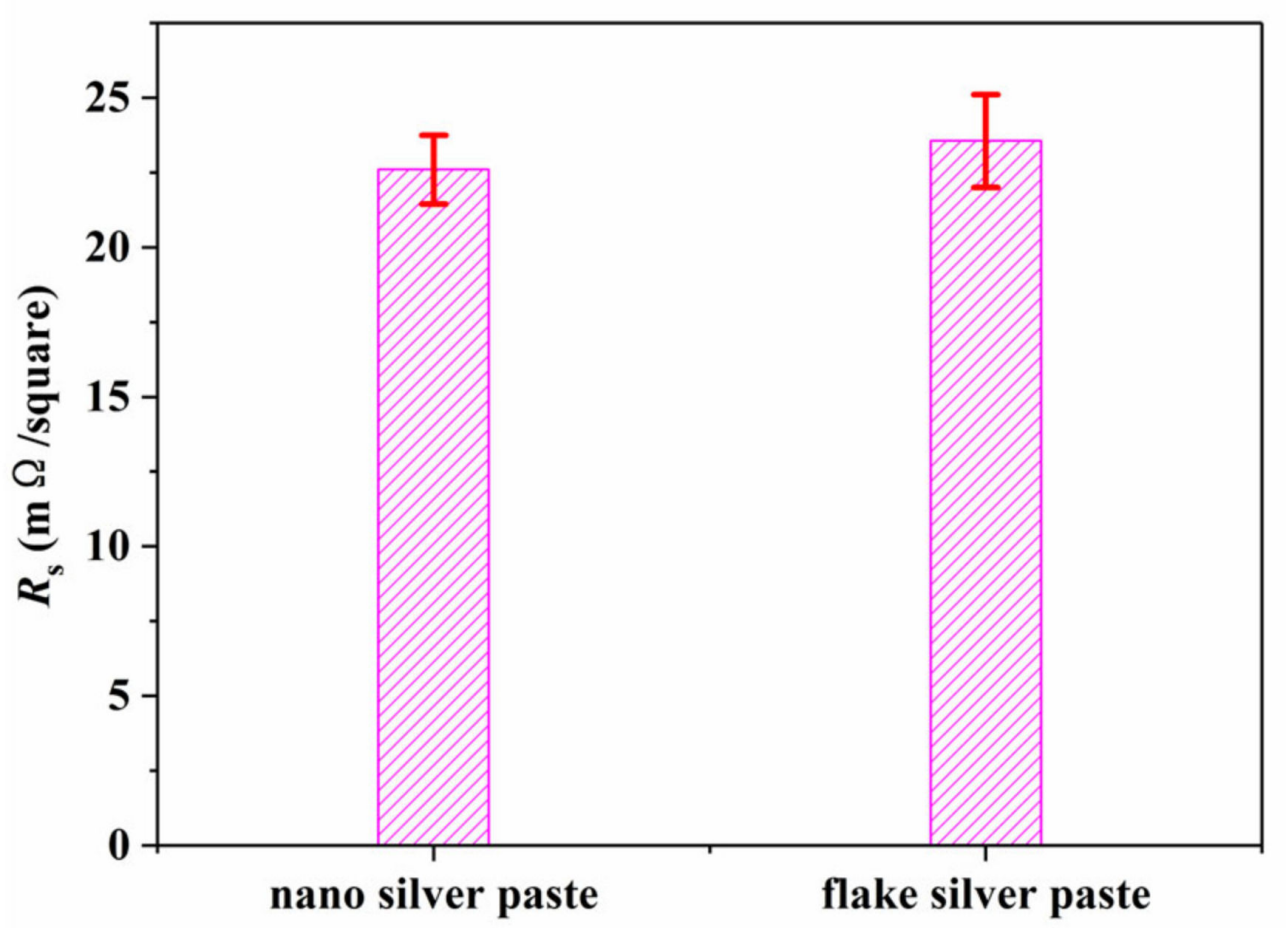
The *R*_s_ of cured nano silver paste and flake silver paste.

In order to elucidate the effect of nano silver powder content on the conductivity of the silver paste, silver pastes with different content of nano silver powder were prepared as shown in Table 1. The conductive patterns were printed on PI film and cured at 300 °C for 60 min. The measured *R*_s_ and calculated *ρ*_v_ is shown in Figure 6. As expected, the *R*_s_ and calculated *ρ*_v_ gradually descend as the nano silver content increases. When the content of silver nanoparticles increases to 83%, the *R*_s_ of the silver paste decreases to 22.6 mΩ/square and remain basically stable with further increase of silver content. It is well known that the conductive network of printed pattern becomes denser as the silver content increases. When the silver content reaches 83%, the density of conductive network in the printed pattern is saturated and the conductivity will not be further improved as the silver content further increase. This explanation can be further confirmed by electron microscopic photographs of conductive patterns as shown in Figure 7. The content of silver and fluorine element in different cured silver paste was measured through energy spectrum and the result was depicted in Figure 7f. As expected, the fluorine content decreases with the increase of silver content. Figure 8 compares the conductivity of silver paste based on flake powder and nano silver powder respectively. It can be found that the silver pastes show the similar *R*_s_ regardless of flake silver powder and nano silver powder, when the mass fraction of silver powder is fixed at 83%. This phenomenon can be explained that the volume of silver powder in the paste has reached saturation, and the dense conductive network has been formed. As is well known, the flake silver powder can provide a larger contact area. However, the nano silver powder has a higher specific surface area, which leads to a higher volume fraction of silver than flake silver powder-based paste at the same mass fraction. Under the combined action of the above two factors, these two silver pastes have similar conductivity.

### 3.3. Rheological Properties

Rheological properties of silver pastes play a key role in determining the performance of the screen-printing process. Silver pastes with accommodative rheological properties will possess excellent screen printable properties, which can contribute to print thinner lines with higher thickness and smoother edges. Moreover, appropriate rheological properties can be conducive to inhibit occurrence of defects and bubbles in the printing process, thereby improving the conductivity of the printed pattern. Two rheological test modes were used to investigate the rheological properties of silver pastes on a parallel plate rheometer.

**Figure 9:**
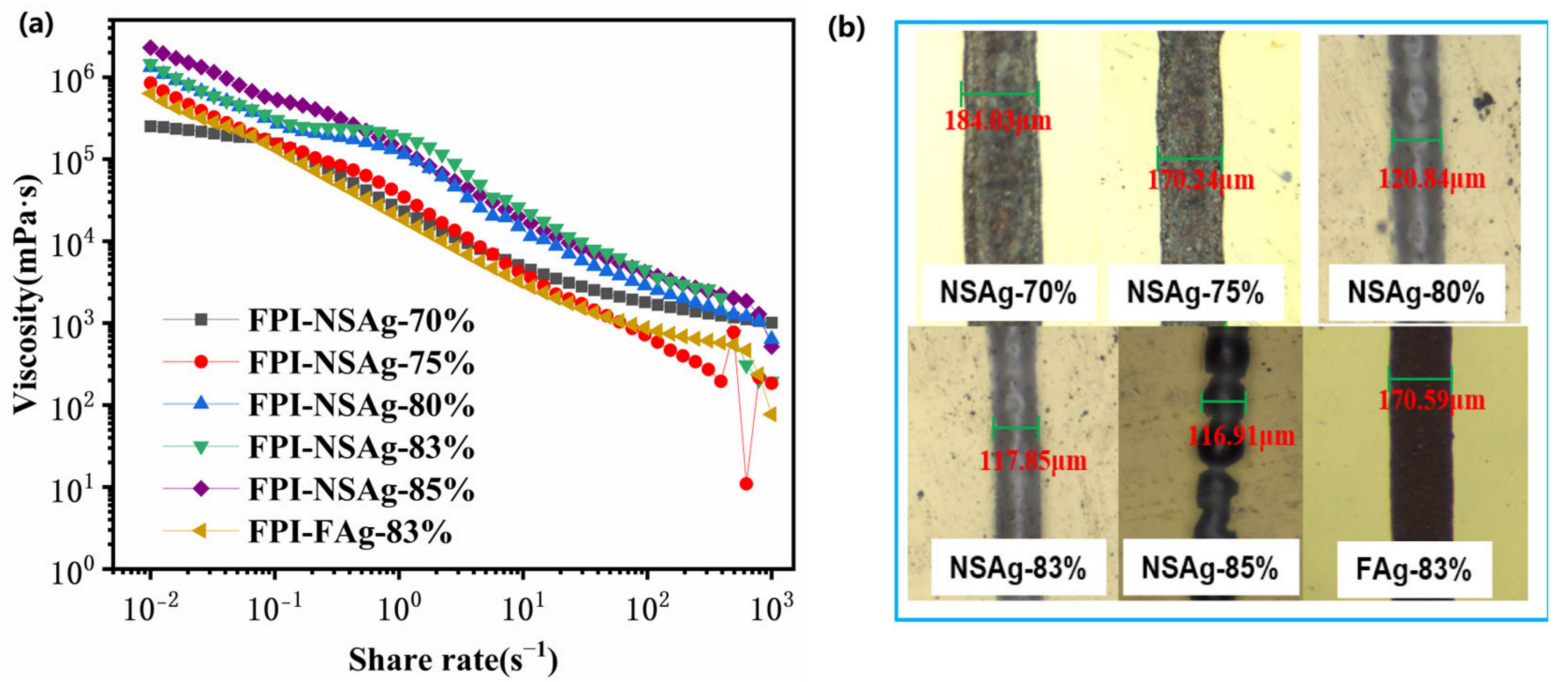
(**a**) The viscosities of nano silver pastes at continuously shear rates. (**b**) The optical micrograph of printed conductive lines.

Figure 9a shows the viscosity of nano silver paste with different silver content at continuously shear rates from 0.01 s^−1^ to 1000 s^−1^. Obviously, the viscosity of all silver pastes decreases gradually with the increase of shear rate, which is consistent with the typical rheological characteristics of pseudoplastic fluids. As the content of silver increases, the viscosity of silver pastes gradually increases, which is due to the reduction of free mobile phase. Figure 9a compares the viscosity shear rate curves of nano silver paste and flake silver paste. It is evident that the nano silver paste possesses a higher viscosity than the flake silver paste; since the nano silver has larger specific surface area, this leads to more resin adsorbed on the surface of nano silver powder. Subsequently, the number of free resin molecules remaining in silver paste is correspondingly reduced. In addition, the high surface energy of nano silver results in graduating of stronger interaction forces in silver paste. All these factors mentioned above will cause higher viscosity of FPI-NSAg paste than FPI-FAg paste. Figure 9b shows the optical micrograph of printed conductive lines with different silver paste. It can be found that printed lines originated from FPI-NSAg-70% and FPI-NSAg-75% show large width of 184 μm and 170 μm, respectively, which are far beyond the design width of 120 μm. This is because viscosities of FPI-NSAg-70% and FPI-NSAg-75% are too low, so that strong flow diffusion occurs after printing on the PI films. Accordingly, as the content of nano silver increases, the printing line width gradually decreases. However, the line printed using FPI-NSAg-85% shows zigzag edges, and the consistency of line width is reduced. This deterioration results from the poor leveling property and high viscosity of FPI-NSAg-85% caused by high silver content. Comparatively speaking, the printed lines from FPI-NSAg-80% and FPI-NSAg-83% show high resolution and smooth edges, the width of printed line is near the design width of 120 μm.

**Figure 10:**
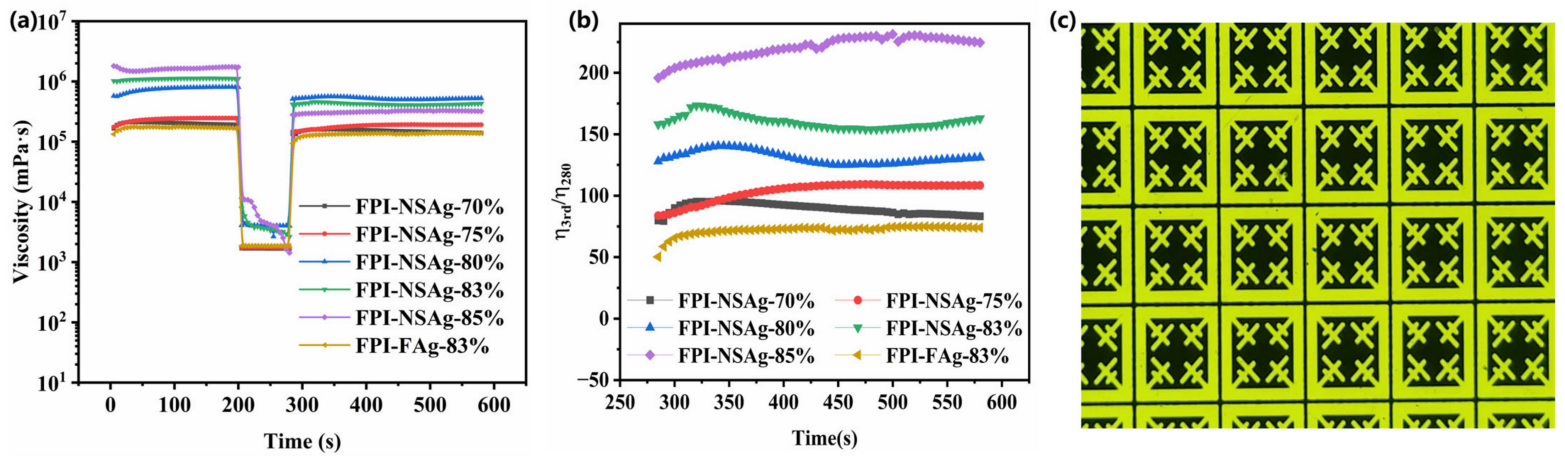
(**a**) The 3ITT curves of silver pastes. (**b**) The viscosity ratio of η_3rd_ to η_280s_ of silver pastes. (**c**) The micrograph of the conductive microstructure of FPI-NSAg-83% paste silk-screened on PI film.

In order to clarify the flow characteristics of silver paste in different stages of the screen-printing process, a three interval thixotropic test (3ITT) was performed with specific test parameters of 0.1 s^−1^ shear rate for 200 s, 100 s^−1^ for 80 s, and 0.1 s^−1^ for 300 s. This test can approximately reflect the rheological changes of silver paste at typical three stages of screen-printing including loading of paste, printing of paste and leaving from silk screen. As shown in Figure 10a, it can be concluded that the viscosity of all silver pastes shows a change of first decreasing and then increasing during a complete 3ITT test process, showing obvious thixotropic characteristics. Moreover, the viscosity of nano paste is higher than the flake silver paste in the first and third stage, which is agreement with the Figure 9a. Generally speaking, the recovery ratio of viscosity in the third stage relates to the first stage, which has a great influence on the size and uniformity of the printing lines, and is usually used to characterize the elasticity of paste [10,20]. However, the prepared silver pastes were partially thrown out during the second stage because of the high shear rate. This can be verified with the decrease of viscosity of paste during the second stage as shown in Figure 10a. It causes a different amount of silver paste between the gaps in the first stage and third stage, so the recovery ratio cannot completely reflect the elasticity. According to the reported literature [10], the viscosity ratio of the third stage (η_3rd_) to the end of the second stage (η_280s_) is used to delineate the elasticity of silver paste. The result is shown in Figure 10b. The η_3rd_/η_280s_ of FPI-NSAg silver paste is higher than that of FPI-FAg silver paste, which indicates nano silver powder is contributes more to enhancing the strength and elasticity of silver paste than flake silver powders. Therefore, the nano silver paste shows an enhanced resistance to strain. Additionally, the prepared nano silver pastes possess outstanding thixotropic characteristics, which can be proved by rapid increase of viscosity after the end of second stage. The good thixotropic property is conducive to improve the printing resolution and obtain printing patterns with high thickness and fine width. Figure 10c shows the micrograph of the conductive microstructure of FPI-NSAg-83% paste silk-screened on PI film. The printed minimum line width and distance are close to 120 μm, which is in accordance with the design size. Additionally, there is no break and dimensional deviation in the entire printed conductive microstructure array. The high dimensional accuracy and smooth edges indicate that the obtained FPI-NSAg-83% paste has good printing adaptability and high printing resolution.

### 3.4. Thermal Endurance

**Figure 11:**
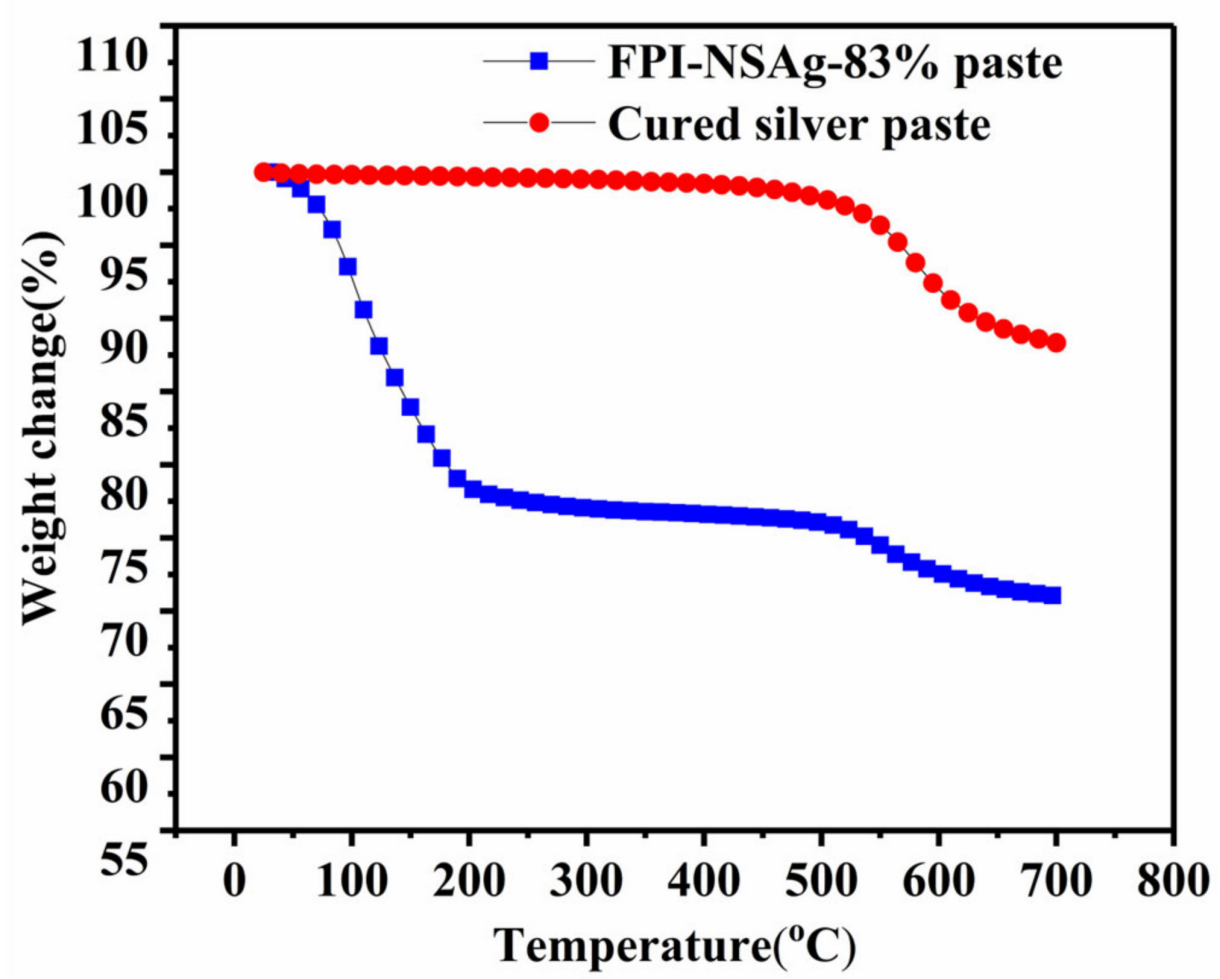
TGAs curves of nano silver paste (FPI-SAg-83%) and cured silver paste under nitrogen atmospheres.

Figure 11 depicts TGAs curve of nano silver paste (FPI-SAg-83%) under nitrogen atmosphere. The thermal weight loss before 300 °C is due to the volatilization of diluent in the paste and the release of water molecule produced by the imidization reaction. This phenomenon further explained the above viewpoint that the appropriate curing time of nano silver paste is 300 °C to obtaining high conductivity. Figure 11 also describes the TGA curve of the cured silver paste (FPI-SAg-83%) under nitrogen atmosphere. It indicates that the obtained nano silver paste shows an outstanding thermal resistance with 5% weight loss temperature higher than 500 °C, which results from the excellent thermal stability of fluorinated polyimide.

### 3.5. Mechanical Properties of Printing Pattern

**Figure 12:**
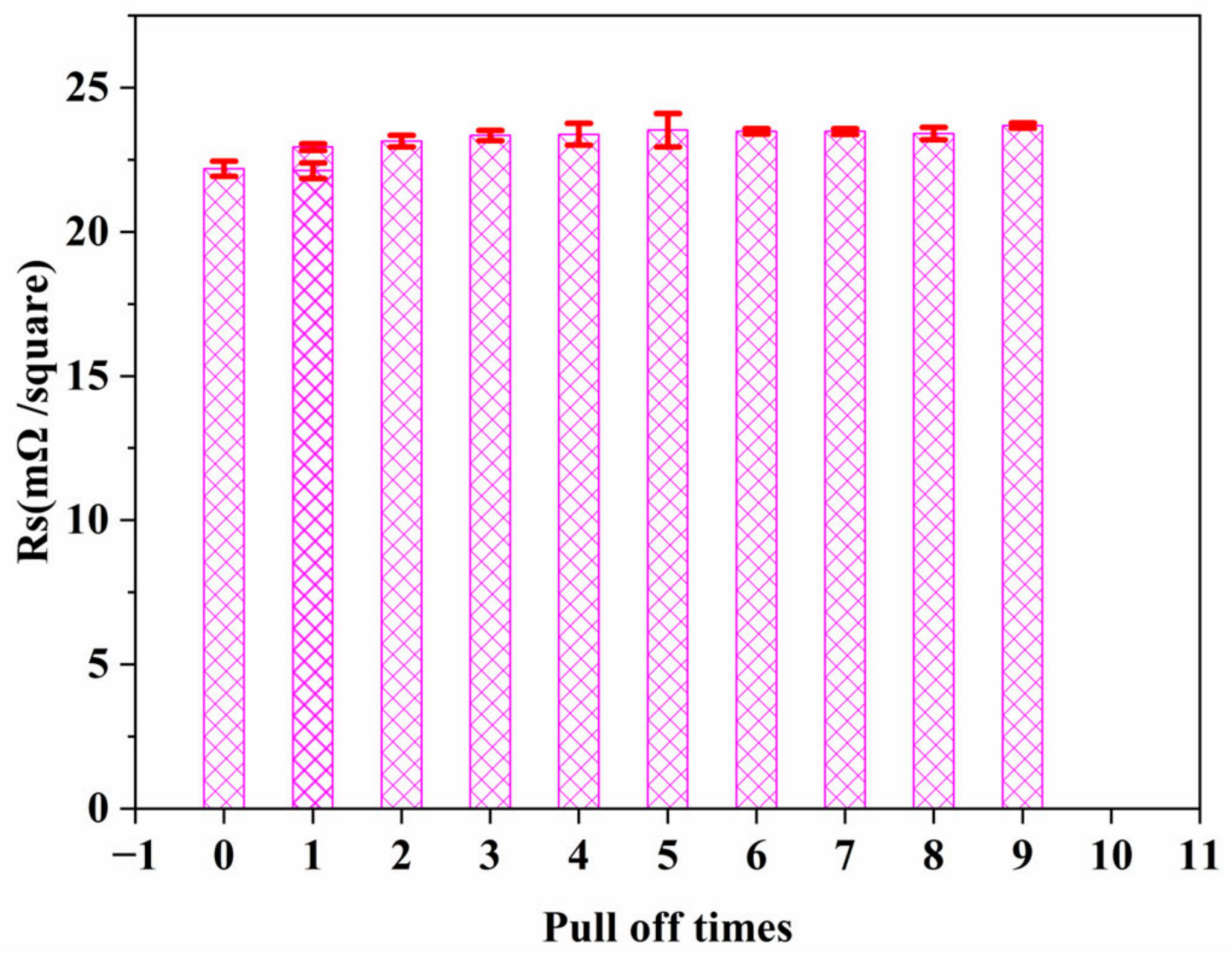
*R*_s_ of conductive pattern after different times peeling off test.

The conductive patterns with dimension of 2 cm × 2 cm were fabricated by screen printing silver paste (FPI-NSAg-83%) on the polyimide films (Kapton-H) through screen printing technology and were further treated at 300 °C for 60 min. In order to evaluate the adhesive strength of conductive patterns and flexible PI substrate, an adhesive tape (3M 600#) was attached to the surface of conductive patterns then peeled quickly at nearly 90°. The *R*_s_ changes after above test were measured using four probe testers, which can reflect the adhesion strength of conductive patterns on PI substrate. Figure 12 shows the *R*_s_ of conductive pattern after different times peeling off test. It can be found that there is no evident reduction of *R*_s_ with the increase of pull-out test times, which means that the conductive pattern has outstanding adhesion strength on the PI film substrate. This mainly due to the good interface compatibility and strong intermolecular force between fluorinated PAA resin and PI substrate.

## 4. Conclusions

In summary, we successfully synthesized fluorinated polyamic acids (FPAA) with excellent solubility in non-amide solvent. The nano silver paste was prepared by mixing the obtained FPAA resin with nano silver powder. The dispersion of nano silver paste was improved by three-roll grinding process with a gap of 5 μm. The obtained nano silver paste possesses an excellent thermal resistance with 5% weight loss temperature higher than 500 °C. The volume resistivity of cured nano silver paste achieves 4.52 × 10^−7^ Ω·m, when the silver content is 83% and the curing temperature is 300 °C. Additionally, the nano silver paste has a thixotropic performance. The printed lines from FPI-NSAg-80% and FPI-NSAg-83% show high resolution and smooth edges and the width of the printed line is near the design width of 120 μm.

## Figures and Tables

**Table 1:** Compositions of the prepared silver pastes.

	Mass of Each Component (g)				
Notes	Nano Silver	Flake Silver	FPAA Resin ^a^	BYK-333	BYK-066N
FPI-NSAg-70%	93.3	0	100	0.97	0.97
FPI-NSAg-75%	120	0	100	1.10	1.10
FPI-NSAg-80%	160.0	0	100	1.30	1.30
FPI-NSAg-83%	195.3	0	100	1.48	1.48
FPI-NSAg-85%	226.7	0	100	1.63	1.63
FPI-FAg-83%	0	195.3	100	1.48	1.48

a: The solid content of FPAA resin is 40%.

## Data Availability

The data presented in this study are available upon request from the corresponding author.
